# Protocol for measuring the effects of cortical scotoma on visual space perception

**DOI:** 10.1371/journal.pone.0352036

**Published:** 2026-11-11

**Authors:** Andrew M. Haun, Elena Monai, Wei Hau Lew, Alex D. Hwang, Giulio Tononi, Karl Friston, Cyriel Pennartz, Melanie Boly, Eli Peli, TWCF-INTREPID consortium

**Affiliations:** 1 Center for Sleep and Consciousness, Department of Psychiatry, University of Wisconsin-Madison, Madison, Wisconsin, United States of America; 2 Department of Neurology, University of Wisconsin-Madison, Madison, Wisconsin, United States of America; 3 Schepens Eye Research Institute, Mass Eye and Ear, Department of Ophthalmology, Harvard Medical School, Boston, Massachusetts, United States of America; 4 Wellcome Trust Centre for Neuroimaging, Institute of Neurology, University College London, London, United Kingdom; 5 Swammerdam Institute for Life Sciences, University of Amsterdam, Amsterdam, the Netherlands; University of Padova, ITALY

## Abstract

We aim to investigate the spatial experience of patients with chronic scotomas caused by lesion to early visual cortex, including primary visual cortex (V1) and adjacent extrastriate visual areas. These experiments are conducted as part of an adversarial collaboration testing contrasting theories of consciousness: Integrated Information Theory (IIT) and two Predictive Processing accounts, Active Inference (AI) and Neurorepresentationalism (NREP). The central question is whether lesions to early visual cortex alter the experienced extent of visual space itself, or instead primarily disrupt stimulus content within an otherwise preserved visual space. To address this, we use paradigms in which patients estimate distances or spatial extents that either span a scotomatous region or fall entirely within intact visual field locations. Psychometric functions relating perceived and physical extent are modeled to estimate shifts in the point of subjective equality (PSE). According to IIT, lesions to early visual cortex, including V1 and occipital extrastriate cortex, should lead to systematic reductions in perceived spatial extent across the scotoma (negative PSE shifts), reflecting a contraction of experienced space. In contrast, Predictive Processing accounts posit that higher-level predictive mechanisms preserve spatial structure despite loss of early input, predicting little or no systematic contraction (with NREP allowing limited context-dependent effects). By quantifying distortions in perceived spatial extent, this protocol aims to distinguish between these competing theoretical predictions.

## Introduction

Damage to early visual cortex can be sufficient to produce homonymous scotomas (the same in both eyes, [1]). Lesions to primary visual cortex (V1) reliably produce complete scotomas affecting all forms of visual sensitivity, while lesions downstream from V1 tend to produce more selective scotomas, such as disruption of color or form perception [2,3] – although certain focal or retinotopically circumscribed extrastriate lesions have been shown to produce scotomas resembling those caused by V1 lesion [4]. In the acute phase (the hours and days after cortical injury), patients generally are anosognosic for cortical scotomas, without any direct awareness of the existence or nature of the visual field defect, even though the deficits may be very extensive (e.g., an entire hemifield may be lost, as in hemianopia) [5,6]. However, patients generally do acquire insight into their condition over time – that is, they come to understand that part of the visual field is lost due to impaired function in daily life [7].

The question of what is happening *phenomenally* in cases of cortical scotoma has theoretical implications that will be investigated in the study described in this protocol paper. Broadly, this has led to two contrasting theoretical perspectives—one in which visual space is actively constructed by higher-level processes, and another in which it is grounded in the intrinsic connectivity of early visual cortex.

### Predictive processing: Visual space as an inferred construct

On some accounts of visual perception, early visual cortex serves primarily as an input layer providing retinotopic information to higher visual areas. In this view, the perception of visual space arises through a constructive process that can be formulated in terms of (unconscious) inference (K. [8–10] or predictive processing (K. [11,12]; C. M. [13–15]. Under these formulations, perceptual content can still be supported by distributed cortical processes beyond early visual cortex that remain functionally intact even after early visual cortical damage. Consequently, when early visual cortex is lesioned, the full visual space may still be represented, but visual stimuli will no longer evoke percepts in the affected region.

In the context of the adversarial collaboration underlying this protocol [16], two variants of predictive processing are considered. The first is Active Inference Theory (AIT; [17–19]), positing that active sampling of the environment (by motor activity such as eye movement or by internalized action such as endogenous attention) is necessary for conscious perception to arise. The second is Neurorepresentationalism (NREP; (C. M. [13,20]), which holds that motor activity is important for consciousness in various ways, but is not strictly essential for it to arise. To enable visuospatial perception and estimation of distances and sizes of objects in visual space, NREP proposes that retinal signals must be integrated with proprioceptive, vestibular, and other sensory or motor-related information to construct a stable spatial representation. This integration is thought to require predictive processing via high-level (e.g., parietal) and low-level (e.g., V1-V2) cortical areas. For example, retinotopic information as represented in V1 needs to be combined with head- and eye position information to achieve a stable visual world representation permitting subjective visual distance estimates (C. M. [20]; C. M. A. [21,22].

### Integrated information theory: Visual space as a retinotopic cortical lattice

The competing view in this project is given by the Integrated Information Theory (IIT) of consciousness [23,24]. According to this account, early visual cortex does not merely contribute input to higher areas, but plays a constitutive role in generating the spatial structure of visual experience itself [25,26]. Specifically, IIT proposes that a two-dimensional lattice of laterally connected neurons (as shown in, e.g., [27–31] – can specify an intrinsic cause-effect structure corresponding to the experience of visual space.

On this view, damage to early visual cortex directly affects the intrinsic cause-effect structure supporting the experienced visual space. When early visual cortex is sufficiently lesioned, the corresponding region of experienced visual space is destroyed. As a result, patients will not report blankness or blurriness in the scotoma region, not because degraded input is being ignored, but because the distinctions and relations that correspond to that part of experienced visual space no longer exist from the intrinsic perspective of the system (absolute agnosia; [32]). In addition, IIT predicts that because the overall composition of the spatial cause-effect structure is changed (with a loss of distinctions and relations), visual space will be distorted beyond the region of visual space where the scotomas are present (affecting subjective distances spanning across them).

### Study objective

The goal of this study is to distinguish between these three competing views by asking patients to directly estimate the amount of space they experience in regions affected by a cortical scotoma. The theoretical logic is straightforward. If visual space is constructed by inference or prediction as supposed by Active Inference, then the lesion of early visual cortex should not change perceived spatial extent. In contrast, if as IIT proposes visual space is specified by the intrinsic connectivity structure of early visual cortex, then lesioning that cortex should reduce the amount of space experienced by the subject—leading to underestimation of spatial quantities across the scotoma. NREP is compatible with a scotoma having no or a small effect, because the overall inference on spatial extent may depend on partial sensory evidence gained from regions directly affected by the scotoma.

To test these predictions, we focus on patients with “convex” scotomas—regions across which spatial judgments can be made. In practice, this includes patients with circumscribed paracentral scotomas and those with quadrantanopic scotomas.

### A perceptual situation to distinguish the alternative accounts

Here, we describe a set of experiments developed as one of four protocols (each published separately; [33–35]) and aimed to test predictions based on different consciousness theories tested in a Templeton WCF adversarial collaboration [16]. To distinguish between the three theoretical accounts described above, we will have patients estimate basic spatial quantities in the context of their cortical scotomas. The stimuli will either span the scotoma or will appear within intact visual field regions.

## Materials and methods

### Eligibility criteria and recruitment

We will recruit broadly, testing any patient whose medical history suggests the presence of a convex scotoma – whose shape allows for stimulus presentation “across” the affected region – due to an occipital cortical lesion. This includes patients with documented paracentral scotomas or quadrantanopia [36]. Paracentral scotomas are of greatest theoretical interest, as they are surrounded by an intact visual field. According to the Integrated Information Theory (IIT) hypothesis, such “holes” in the field could potentially yield more pronounced effects than quadrantanopias or hemianopias, since they amount to a qualitative change in the topology of the visual experience. We will not include patients with hemianopia, where the scotoma occupies most or all of a hemifield, as it is not possible to place stimuli that span the scotoma in these cases.

At University of Wisconsin–Madison participants will be identified through clinical databases and through the Neurology and Ophthalmology departments, based on documented history of scotoma or quadrantanopia. When available, clinical neuroimaging (CT or MRI) will be reviewed to confirm lesion location, and clinical perimetry will be used to determine eligible visual field defects. At the Schepens Eye Research Institute (SERI, Boston), participants will be recruited through the Peli Lab patient database of individuals who had previously participated in studies conducted at various SERI laboratories, and the Research Patient Data Registry (RPDR) of Mass General Brigham. SERI lab personnel will also review clinical neuroimaging when available, and will recruit participants based on appropriate visual field loss profile (quadrantanopias, paracentral scotomas, or other convex field scotomas). This is an observational psychophysical study and does not constitute a clinical trial, as no intervention is assigned and no health-related outcome is evaluated. Both UW–Madison and SERI have received Institutional Review Board (IRB) approval for the study (UW-Madison HS-IRB 2018-0320, and MGB IRB protocol 2022P003101, respectively), and written consent will be obtained from all subjects before their participation. According to both SERI and UW-M IRB protocols for testing adult subjects, participants must be between 18 and 85 years of age, capable of providing written informed consent, and able to comply with experimental procedures. Any prior participation in vision studies or visual rehabilitation programs will be recorded for each subject, as will other potentially-relevant individual factors beyond simple demographics, such as time since injury and concomitant neurological or visual issues in addition to the cortical visual field defect. (None of these factors were noted in the preregistration as having any particular significance).

The recruitment goal for each group is 24 patients, with a similar number of control subjects against which to calibrate the patient data. This number was set after early discussions of feasible recruitment over the 2–3 year duration of the project based on discussions with clinical collaborators at both sites.

### Experiment apparatus

In the UW lab, all experiments and perimetry will be performed using code written in MATLAB using the Psychophysics Toolbox, run on a Windows PC. The experimental display at UW is a Samsung C49G9 wide-field monitor (114.8 cm wide, 53.6 cm high) that allows for large horizontal stimulus extent. This is viewed at a distance of 65 cm, allowing for a horizontal/vertical field of view of about 105 degrees wide and 30 degrees high. The SERI group will use their own version of the experiment code written in Unity to integrate with their own perimetry routines, with stimuli viewed on a 75” TV display (Jung et al., 2022; [37]) at a distance of 64 cm, allowing a horizontal/vertical field of view of 104 degrees wide and 71 degrees high. While these are large-field displays, it is noteworthy that both setups do allow full visibility of the rectangular limits of the displays.

### Perimetry, stimulus placement, and testing

The first phase of the study is screening of the extent and location of each participant’s scotoma at the time of testing. Initial confirmation of the scotoma is obtained through a confrontation visual field test. The participant is then seated in front of the experiment display in a dimly-lit room. The experimenter performs a brief calibration of the EyeLink 1000 eye tracker to ensure accurate fixation tracking. Each participant is identified by a unique code number, which is used to label all saved data and maintain anonymity.

The fixation point on the display is positioned based on the approximate location of the scotoma. For example, if the scotoma is located in the lower right visual field, the fixation marker is placed nearer to the top of the screen, ensuring that more of the scotoma falls within a testable region of the display area. Visual field mapping is performed using a custom perimetry routine and proceeds in two stages: *static* and *dynamic* perimetry. During both perimetry phases and during the following stimulus placement phase, the screen is filled with a gray background color, and the fixation mark is a bright yellow dot 0.5° in diameter. During the experiment tests, the display is filled with a black background color, and the fixation mark is a 0.5°x0.5° white square. Fixation data will be recorded using an Eyelink 1000 eye tracking system (Smart Eye Pro Version 6.1, Goteborg, Sweden, 2015 for SERI lab) through all phases of the study. Fixation is monitored in real time by the experimenter: if participants are observed to frequently look away from the fixation marker, they will be reminded by the experimenter of the importance of fixating the marker. Because the critical stimuli are placed relative to the participant’s mapped scotoma, systematic fixation offsets could in principle shift stimuli relative to the intended retinal locations, and we therefore treat gross fixation instability as a *post hoc* exclusion criterion (completing data collection with a subject regardless of observed fixation failures). If a participant’s within-interval fixation deviates egregiously (more than 2°, i.e., well outside the fixation box) on a majority of trials, that participant shall be excluded from final data analyses – otherwise we will provide fixation metrics as part of the general results.

For static perimetry (Fig 1A), the participant maintains fixation on a yellow dot. The experimenter views a separate control display showing a schematic representation of the participant’s visual field and moves a hidden cursor to various test locations. At each location, the experimenter triggers the appearance of a probe stimulus: a high-contrast (98% Michelson contrast) 2 × 2 checkerboard pattern that counter-phases at 4 Hz and defaults to 0.5° visual angle in width (2 cycles per degree). The experimenter queries the participant verbally (“do you see the probe?”). The participant verbally responds “yes” or “no” to indicate whether the probe was seen.

**Fig 1:**
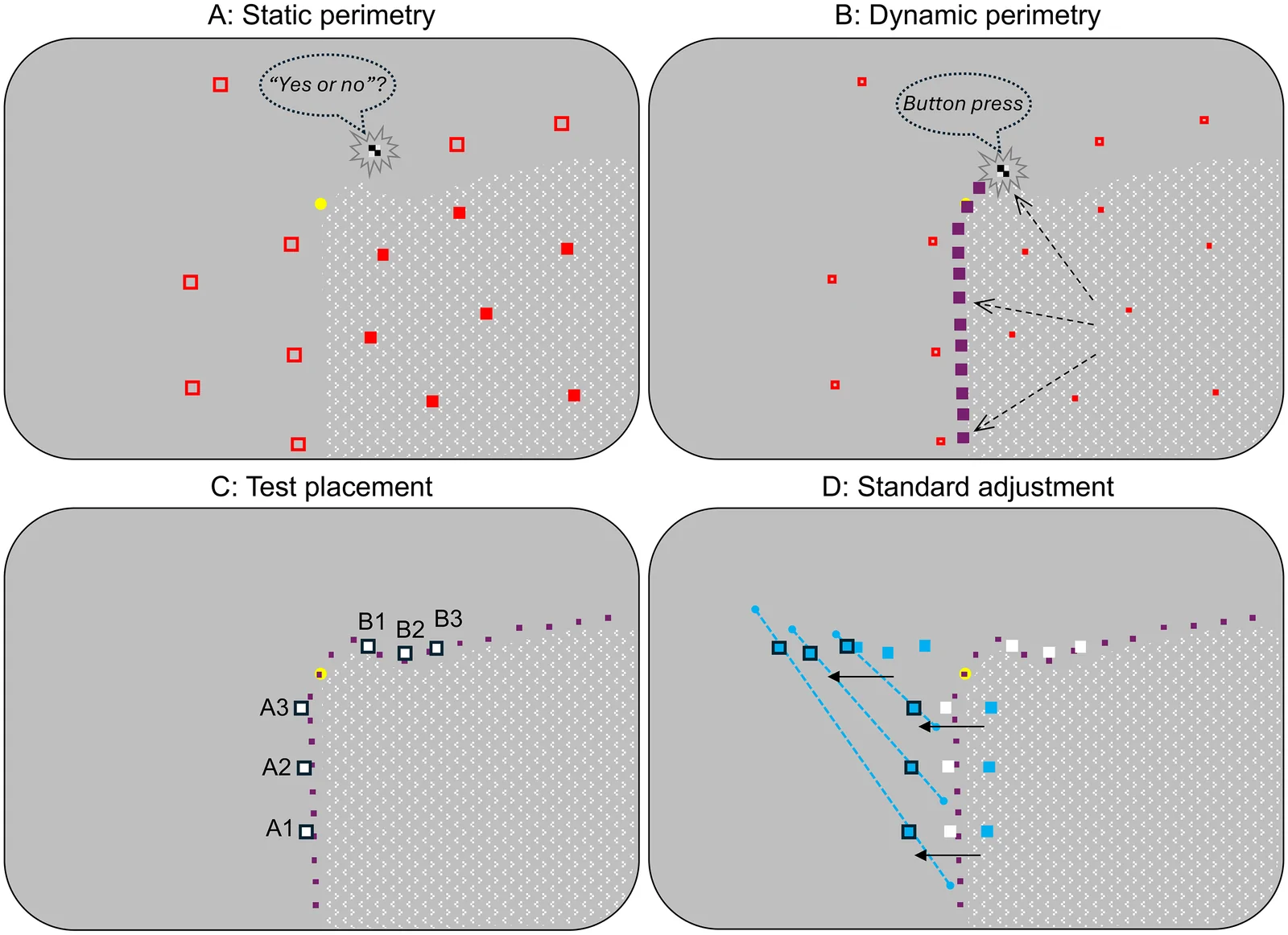
Perimetry and stimulus setup for a right inferior quadrantanopia. A) First, a coarse map of the scotoma is established by a low resolution static perimetry procedure. The experimenter presents the flashing checkerboard probe while the participant fixates the yellow dot, asking “do you see the probe – yes or no?” The participant responds verbally and the experimenter records the response (here shown as empty/solid red markers). The map of responses is visible only to the experimenter. B) Starting from within the coarse scotoma, the experimenter gradually moves the probe toward the seeing field. At first sight of the probe, the participant presses a button. A sequence of these probes reveals the fine boundary of the scotoma (purple markers). C) The experimenter places six test positions (white markers) outside the scotoma border: the first three are the ‘A’ group and the other three are the ‘B’ group. D) The normal-field standard positions are set initially as a copy of the test positions flipped left-right across the vertical midline (edgeless blue squares). The experimenter then adjusts the standard array (illustrated by the black arrows) so that expanded positions will not fall into the scotoma, or off the display bounds (blue dashed lines show a subset of the maximally expanded standard stimulus positions).

Perimetrically-defined defects are frequently non-uniform, and vision loss can be graded, especially at the boundaries of a scotoma, with some regions retaining degraded or fluctuating residual function rather than dense blindness [33,38]. Because patients with cortical field loss also vary in how readily they interpret indistinct percepts, the response criterion is established explicitly with each participant through brief interaction: participants are asked to respond when they see something at a definite position, i.e., a percept they could localize or point to, rather than a vague or unlocalized impression of seeing, so as to isolate regions of dense visual loss. If no clearly non-responsive region is found but an area of degraded vision is amenable to testing, the criterion is shifted, again through interaction, to responding only when the target is seen clearly (appearing as a something like the checkerboard probe). The operative criterion is confirmed with each participant before mapping and noted for that session, and the experimenter records each response to produce a coarse binary map of visibility across the field.

Once the coarse layout of the scotoma is established with no more than 10 probe presentations, the experimenter begins dynamic perimetry (Fig 1B). The participant continues to maintain fixation while the experimenter presents the same checkerboard probe within the blind field and slowly moves it toward the seeing field. (The probe motion is not strictly controlled, but at a gradual pace, amounts to several degrees per second). The participant holds a button box and is instructed to press a button as soon as the probe becomes visible, applying the same response criterion established during static perimetry. This procedure yields a map of the boundary between the blind and seeing regions, with a bias toward positions *outside* the scotoma region (since the participant responds after the target has appeared, not when it disappears). The experimenter’s goal is to outline the scotoma so as to capture bends and prominences on the scale of a few degrees across – for a typical quadrantanope, 20 positions should suffice for the requisite resolution.

Stimulus test locations are determined interactively for each participant based on their mapped scotoma (Fig 1C). While the participant maintains central fixation, the experimenter references the perimetry data on the secondary monitor and selects candidate positions for testing. Three positions are assigned to one “side” of the scotoma (Group A) and three to the opposite side (Group B): it should be the case that any pair of points drawn one from A and one from B should span the scotoma. At each chosen location, the standard checkerboard stimulus is presented and the participant is asked to confirm clear visibility. If the participant reports any ambiguity—such as blur, low contrast, or delayed detection—the experimenter shifts the position slightly away from the scotoma boundary and repeats the visibility check until the stimulus is confidently seen. We note here that because a typical quadrantanopic defect abuts the vertical meridian, configurations spanning the scotoma must cross into the intact hemifield, while the intact-field comparison stimulus will be contained entirely within that hemifield. In cases of paracentral scotoma where perfectly symmetrical test/standard configurations are possible, they will be used preferentially.

By design, the scotoma is bracketed between Groups A and B, ensuring that all six test positions lie in intact field regions on either side of the scotoma. Once these test positions are finalized, the software generates a corresponding set of control (“standard”) positions by reflecting the tests across the vertical meridian into the opposite hemifield (Fig 1D), also indicating the range of possible contractions or expansions of the control positions. The experimenter verifies that the standard stimuli will fall in visible regions of the display. If any positions fall within the scotoma or outside the display, the entire set of standard positions can be shifted together to a nearby visible region (the distance of the shift is indicated on the experimenter’s display) while preserving their relative spacing. This procedure ensures that test and control stimuli are presented in comparable, reliably visible locations.

Adjustment of stimulus distance or size is the same for all three tasks. The standard stimuli are ‘expanded’ or ‘contracted’ around their mean position, which does not change. That is, stimulus positions are adjusted by the parameter δ by simple affine scaling:


$$\begin{array}{c} x_{standard}=\delta \left(x_{default}-\overset{―}{x}\right)+\overset{―}{x} \end{array}$$ (Eq.1)

The scaling parameter δ is fixed to a frame ranging from 0.5 to 1.5, in steps of 0.1.

### Procedure

We will test subjects on up to three tasks, each involving estimation of the spatial extent of a visual stimului. Each task uses a two-interval forced choice (2IFC) design (Fig 2): on each trial, two stimuli are shown in sequence, and the participant indicates which one appears larger, spans a greater distance, or has parts that are further apart. Instructions emphasize that participants may rely on whichever subjective impression makes the most sense to them. Responses are recorded via a hand-held button box.

**Fig 2:**
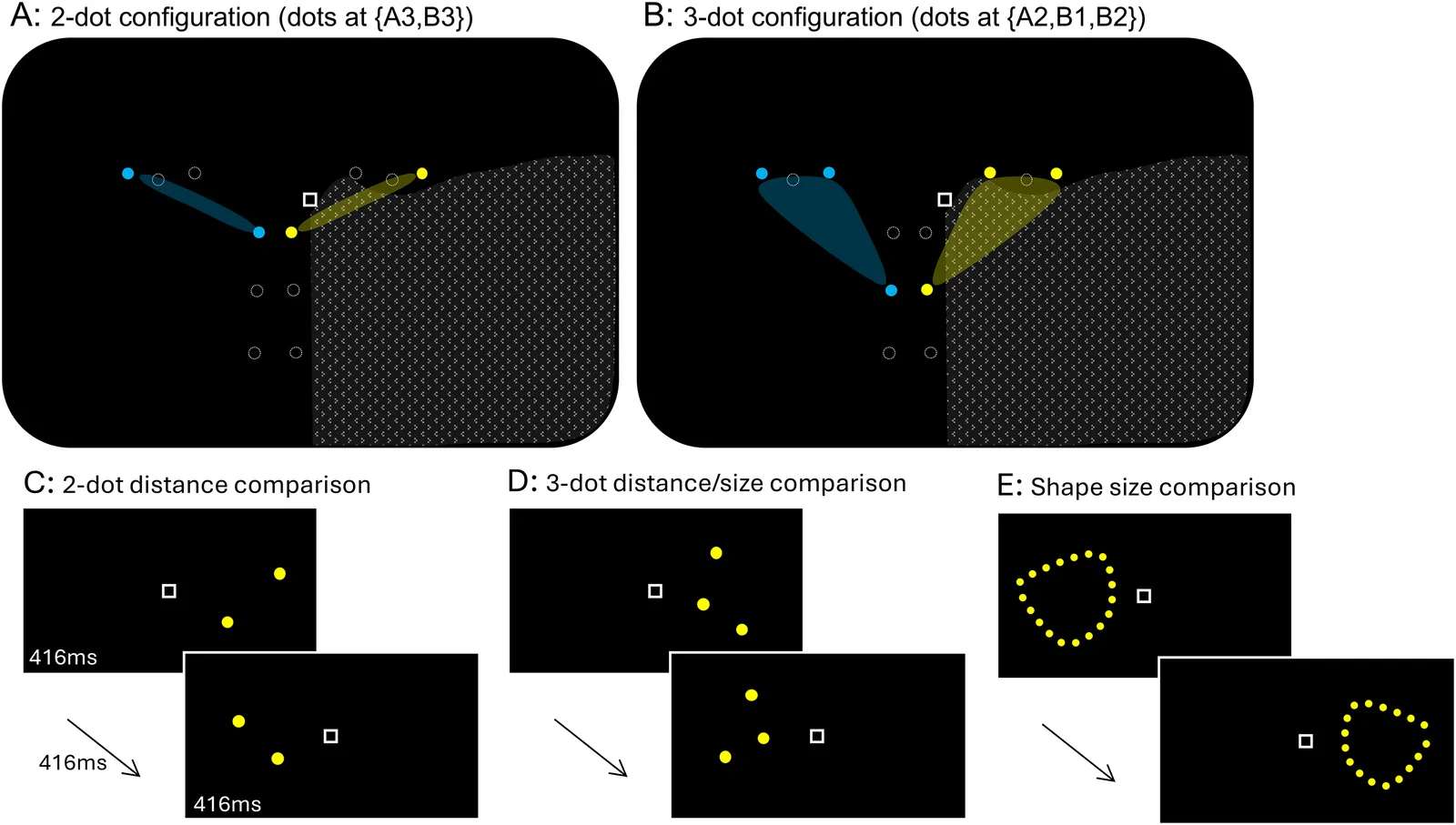
A,B) Example stimulus positions based on those in Fig 1. White squares indicate the fixation marker and the stippled region represents a scotoma. Yellow dots indicate test and standard positions. The colored regions suggest the parts of space for which the stimuli are used as a basis for subjective estimates. A) On this 2-dot trial, the stimulus positions are at A3 and B3. B) On this 3-dot trial, the stimulus positions are at A2, B1, and B3. C,D,E) Schematic illustrations of the 2-interval forced choice (2IFC) task. The participant must decide which stimulus, the first or the second, appeared to cover a larger spatial extent (i.e., distance (C) or area (D,E)).

Each pair of stimuli consists of a “test” (spanning the scotoma) and a “standard” (presented in the field), arranged as mirror-symmetric pairs around the vertical midline modulo any positional adjustments determined in the placement phase (Fig 1D). On each trial, the test stimulus appears in one of the pre-defined test positions, sampled in pseudo-random order to avoid repetition and to allow separate (though lower-precision) estimates of each test location’s apparent span. The standard positions are adjusted trial-by-trial according to 1-up 1-down staircases, which target the Point of Subjective Equality (PSE)—the point at which the participant is equally likely to choose either interval. The order of test/standard on each 2IFC trial is pre-randomized, so half of the trials are test/standard and half are standard/test.

During a block of trials, there may be 2 or 4 interleaved staircases, each running for a fixed number of trials (depending on the experiment, as described below), with starting points well above or below the point of physical equality so that the participant should be making ‘easier’ choices at the beginning of each block. The number of staircases can be adjusted to shorten or lengthen the block, depending on participant stamina or other considerations. Each staircase updates the standard stimulus using a 1-up 1-down rule, with 20% step sizes. However, the staircases are offset so that their values interleave, effectively sampling from a shared underlying set of size levels spaced at 10% intervals. This creates a fixed-resolution but adaptively sampled stimulus set, allowing for efficient estimation of the PSE [39].

At the end of each block, the experimenter reviews a summary plot showing trial distributions and the emerging psychometric function. If ceiling or floor cases are encountered, where a participant seems to have an effect large enough that it exceeds the frame, the experimenter can substitute a new frame (shifted to larger or smaller values) to be used on subsequent blocks. Data are considered acceptable if the function clearly shows a monotonic increase in proportion of “the standard was larger” responses with increasing standard/test ratios, regardless of PSE location – such a trend should be easily visible with as few as 24 trials. If results deviate from this pattern (the trend may be reversed, indicating inadvertent button-swapping, or flat, indicating either misunderstanding of the task or inability to see the stimuli), the experimenter will review instructions with the participant to determine the problem. Participants will complete a minimum of four blocks for each task (154 two-dot trials, 96 three-dot trials, and 96 contour-shape trials). More blocks should be added only if time permits – blocks should *not* be added on the basis of the pattern of results.

All participants will run the two-dot and three-dot tasks (Fig 2C,2D). The contour-shape (Fig 2E) task is reserved for clear cases of paracentral scotoma, where the stimulus can be certain to fully surround the scotoma.

### Stimulus conditions

#### Task 1: Two-dot distance estimation.

The two-dot task indirectly measures perceived distance across a scotoma by comparing a pair of dots placed on either side of the scotoma (the *test* stimulus) with a pair of staircase-adjusted dots in the unaffected hemifield (the *standard* stimulus). Each dot is a bright yellow disk 1° in diameter.

Participants maintain fixation on a white square throughout the block. On each trial, one pair of dots (either test or standard) is shown for 416 ms, followed by a 416 ms interstimulus interval (ISI), and then the second pair for another 416 ms. The first and second intervals are accompanied by low (400 Hz) and high (800 Hz) tones. Participants press one of two buttons to indicate which pair of dots appeared further apart, the first or the second. Participants will complete a minimum number (~10) of practice trials to confirm understanding before data collection begins.

Test dot pairs are formed by selecting one dot from Group A and one from Group B (see Stimulus Placement and Fig 2A). Over the course of a block, each possible A–B pairing (9 total) appears equally often within a staircase. With the default of 2 repetitions per pairing, each staircase runs for 18 trials. Half the trials begin with the test pair and half with the standard, randomized in advance. Longer blocks can be created by increasing the number of repetitions (e.g., to 3 or 4) or the number of staircases (e.g., from 2 to 4). The full trial order is randomized before the block begins.

#### Task 2: Three-dot distance estimation.

The three-dot task is very similar to the two-dot task. It indirectly measures perceived distance across a scotoma by having participants compare configurations of three dots that span the scotoma with corresponding configurations in the intact visual field. Participants may base their judgments on the mutual distances between dots, on the overall size or extent of the triangular configuration, or some combination of these cues. The test and standard dots are yellow 1° disks as in the two-dot task. Timing, fixation, and response parameters are the same as in the two-dot task.

The test dots are chosen so that two of the dots are at the ‘outer’ two positions from either Group A or Group B (i.e., A1 and A3, or B1 and B3; see Fig 2B), and the third dot is one of the three positions from the other group, yielding 6 possible dot triplets (Fig 2B). As with the two-dot task, each pairing appears the same number of times within each staircase. With 2 repeats per triplet, a staircase runs for 12 trials. Longer blocks can be created by increasing repeats or staircases, and other counterbalancing follows the same logic as in the two-dot task.

#### Task 3: Contour size estimation.

The third task uses a long string of dots (the ‘contour’) surrounding the scotoma as a test stimulus, with a reflected copy of the contour, adjustable by staircase, in the normal field. This task is reserved for participants whose scotoma is a ‘hole’ in the visual field, with a functioning visual field in every direction outward from the scotoma.

The contour is defined by fitting an ellipse using a least-squares method to the six test locations used in the 2- and 3-dot tasks, and adding a low-frequency noise modulation to the ellipse to produce a randomizable ‘blob’ shape that follows, but will always exceed, the outer boundary of the scotoma: the addition to the fitted ellipse is always at least +10% of the ellipse radius at a given point, and no more than +40%, so the test points (and thus the scotoma itself) will always be *within* the bounds of the shape. The shape is regenerated on each trial. The standard contour size is adjusted by 2 interleaved staircases – no other parameters are varied. Each staircase runs for 12 trials, with longer blocks (e.g., 24 or 36 trials per staircase) used when appropriate. As in the other tasks, the participant judges which shape—first or second interval—appeared larger.

## Data analysis

### PSE estimation

The simple experiment design—1-up 1-down staircases—is intended to yield a straightforward measure of relative perceived distance by estimating the *Point of Subjective Equality* (PSE) between scotomatous and normal regions of the visual field. PSEs are estimated by fitting psychometric functions to trial data using maximum likelihood estimation (MLE), with bootstrapping used to determine the precision of these estimates (Wichmann & Hill, 2001a; [40], 2001b; [41]). Specifically, we fit a logistic function of the staircase-driven stimulus scaling parameter δ (Eq 1) with an unbounded search over three free parameters: the mean ($\delta_{PSE}$), corresponding to the PSE; the slope (β), reflecting sensitivity to changes in δ; and the lapse rate (γ), capturing the frequency of stimulus-independent errors such as attentional lapses or button-press mistakes:


$$\begin{array}{c} \psi (\delta )=\gamma +\frac{\left(\text{1}-\text{2}\gamma \right)}{\text{1}+exp\;\left(-\beta \left(\delta -\delta_{PSE}\right)\right)\;} \end{array}$$ (Eq 2)

The primary effects of interest are shifts in the PSE away from the *Point of Physical Equality* (PPE). In the absence of any scotoma-induced distortions, the PSE is expected to coincide with the PPE.

The spread of recovered PSEs across these repetitions (Fig 3C) provides a demonstration of the method’s sensitivity. For an observer with low lapse rate (γ = 1%) and moderate sensitivity to changes in δ (β = 0.1, i.e., a ‘discrimination threshold’ of about 10%), the standard deviation of the test distribution over a 300 trial experiment is about 0.038, so an effect as small as δ=±0.063 (z_0.95_σ ≈ 1.645 × 0.038 = 0.063) would be detected in 95% of cases. This precision is approximately independent of the magnitude of the true shift within the range of the staircase frame, but depends on the participant’s lapse rate and psychometric slope, and on the number of trials completed. We therefore do not assume a fixed sensitivity across participants; the precision achieved for each participant will be inferred on an individual basis (e.g., by bootstrapped confidence intervals or model selection methods).

**Fig 3:**
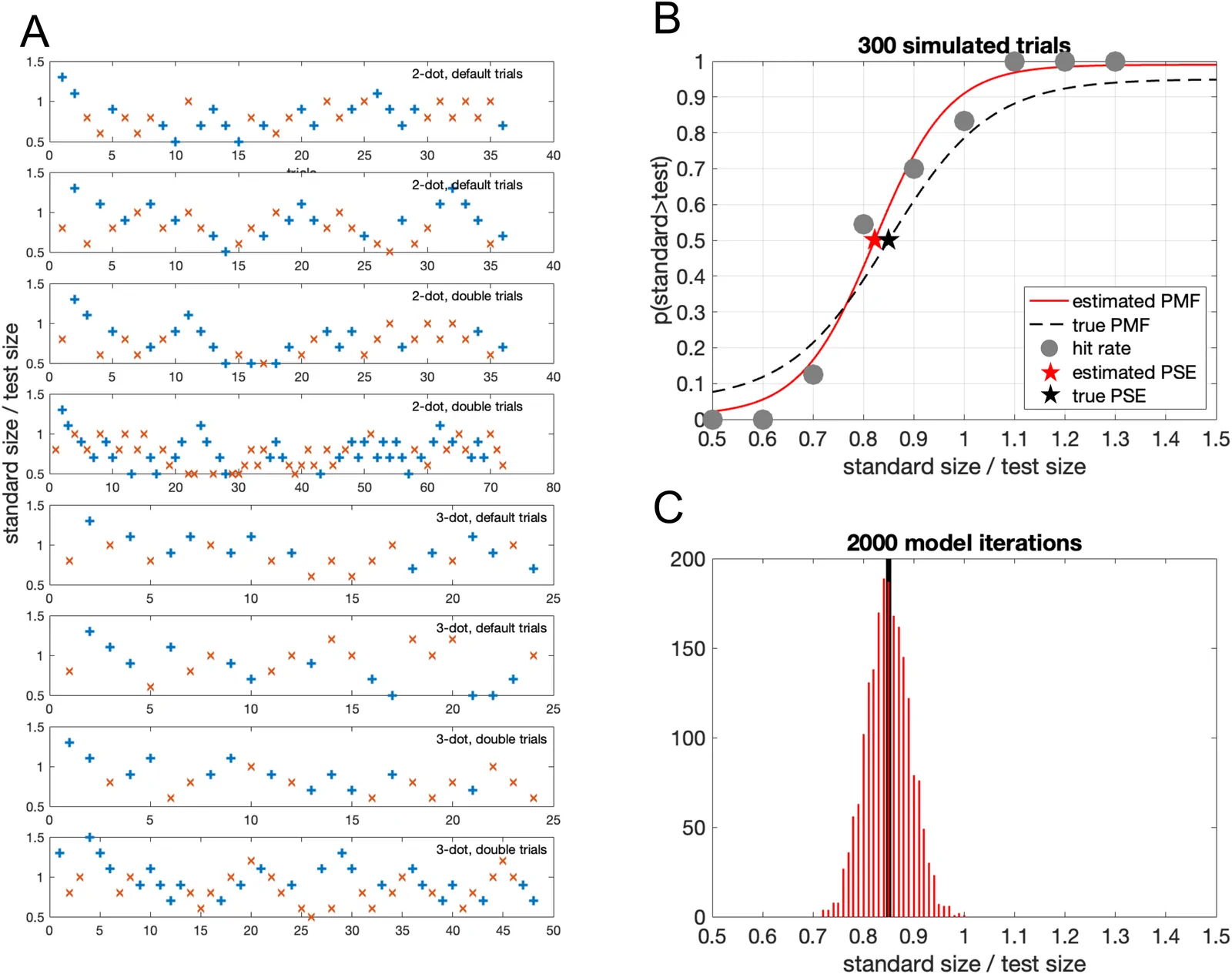
Simulated data collection and analysis to demonstrate the precision and accuracy of the measurement technique. A) In this scenario, the (simulated) participant runs through two tasks: 4 blocks of the 2-dot task, and 4 blocks of the 3-dot task. In each task, the fourth block has twice the number of trials, simulating a case where the subject seems to have stamina for a longer block. Red and blue symbols indicate the different interleaved staircases. B) The data are pooled across all blocks of both tasks to construct a psychometric function (PMF), which is fit with a curve (red line) to estimate the point of subjective equality (red star, PSE). In this case, this may be compared with the ‘true PSE’ (black star) that was used to define the model a priori. C) This experiment scenario was repeated 2000 times to yield a distribution of estimated PSEs for this simulated observer.

### Connecting results to the adversarial predictions

Integrated Information Theory (IIT) predicts a systematic negative shift in the PSE: if visual space is severely reduced or absent within a scotoma, then a physically smaller standard stimulus should appear to match the extent of the test stimulus (i.e., PSE < PPE). By contrast, Active Inference Theory (PP-AI) predicts no systematic shift (i.e., PSE = PPE), as perceived spatial extent is expected to be maintained via inference based on intact visual information. Neurorepresentationalism (PP-NREP) predicts either no shift or a modest negative shift in PSE, reflecting partial reliance on information spanning the scotoma in combination with intact visual input. For each participant, the analysis first asks whether the data support any departure of the PSE from physical equality. This is assessed using standard model-comparison methods (for example, comparing a shift-inclusive psychometric model against a reduced no-shift model by an information criterion such as AIC [42]) to determine whether a PSE shift is supported by the data collected. This yields a per-participant classification: evidence for a negative (contractive) shift, evidence for no shift, or evidence too weak to distinguish these. Because the theories make claims about the structure of individual experience, they are adjudicated at the level of the resulting distribution across participants rather than by a single pooled test. A preponderance of supported negative shifts would favor IIT; an absence of supported shifts in most participants would favor Active Inference; and an intermediate pattern – shifts in some participants but not others – would be most consistent with Neurorepresentationalism. Any preponderance of positive ‘expansive’ PSE shifts would constitute an outcome predicted by none of the three accounts.

By design, there are several levels at which PSEs can be measured, within a single participant, from the data:

1. *Global level*: All data across tasks within a single participant are pooled, under the assumption that the underlying effect is robust and relatively task-independent. Since all tasks use the same 2IFC paradigm and the same scaling parameter, data can be combined straightforwardly (pooling all trials for each δ level).
2. *Task level*: PSEs are estimated separately for each task, allowing for possible differences in effect size or direction across task types. Multiple tasks were included in the protocol in part because they may differ in sensitivity to the effect, so that a shift detectable in one task may be attenuated in another; the global pool nonetheless remains the primary outcome, as the preregistration made no predictions distinguishing the tasks.
3. *Stimulus-specific level*: PSE shifts are examined in relation to specific stimulus features such as visual field angle and eccentricity, scotoma size or shape, and span direction across the scotoma.

In addition, at the finest grain, it is possible that trial-to-trial judgments about specific stimuli may be meaningfully analyzed, and so all stimulus data is retained for all trials in each experiment. It should be noted that the INTREPID preregistration made no specific predictions at the task or stimulus-feature level, and so the global PSE measure should be treated as the primary outcome of interest: the model-comparison classification described above will be applied to this global per-participant PSE. The per-participant classification and its use to adjudicate the theories is a pre-specified interpretive procedure described here, though not itself part of the original preregistration. Other analyses may provide useful insights into the factors modulating the perceptual consequences of scotomas.

PSE shifts estimated in this way offer the most direct view of the experimental outcomes. More sophisticated models may be developed—accounting for performance across participants as a function of scotoma geometry or the specific visual areas affected by the lesion—though the substantial variability expected across individuals may make this difficult. Lesion locations will differ markedly, and scotomas will take on idiosyncratic forms. For this reason, the initial analyses should focus on global-level measures on individual participants, with data combination guided by lesion characteristics, providing a clearer picture of perceptual changes on a case-by-case basis. This case-by-case emphasis is the reason the theories are adjudicated over the distribution of per-participant outcomes, as described above, rather than by pooling across participants into a single test.

## Discussion

This study protocol describes a set of related experiments designed to measure distortions in visual space that may arise following lesions to occipital visual cortex. The study was proposed and developed as part of of an adversarial collaboration (INTREPID, Templeton World Charities Foundation; https://arc-intrepid.com/) involving proponents of three theories of consciousness: two predictive processing-based accounts – active inference (AI) (K. J. [43] and neurorepresentationalism (NREP) (C. M. [13], and integrated information theory (IIT) [23]. In a pre-registered document, these theories articulated different predictions regarding the expected pattern of results [16].

As outlined in the Data Analysis section, the interpretation of results from these experiments will rely on observed shifts in the PSE due to scotoma-crossing stimuli. Consistent negative PSE shifts would support the predictions of IIT and, to a lesser extent, NREP, whereas the absence of systematic shifts would support the predictions of AI and, to a lesser degree, NREP predictions). To be clear, the strength of support depends *also* on the strength of the theoretical predictions [16,44]. No theory predicted consistent **positive** PSE shifts.

Perceptual distortions in the vicinity of scotomas and blind spots are not unheard of, and have been reported under several circumstances. Shape distortions of objects extending into visual field defects have been described [45,46]. Even “artificial scotomas” in normally sighted observers can produce transient spatial distortions under certain circumstances (e.g., Tailby & Metha, 2004; [47]). Distortions associated with paracentral scotomas have also been documented clinically, including the “thin man” phenomenon, in which stimuli spanning the defect appear compressed [48–50]. To our knowledge, however, the latter work represents the only prior attempt to directly assess the apparent extent of space spanning a cortical scotoma, and it did so in two patients, using a single stimulus configuration (continuous vertical lines) and only qualitative or quasi-psychophysical methods. Our aim here is to characterize any effect quantitatively and psychophysically across a larger sample and a range of stimulus configurations. We expect that such a broader sample of cortical scotomas may raise challenges due to the inevitable heterogeneity of scotoma etiologies and presentations.

### Status and timeline

The experimental paradigm reported here was developed as part of the INTREPID adversarial collaboration, following consortium workshops and coordinated protocol design across theory teams. Initial piloting and task development were conducted in 2022, with formal data collection at the primary site beginning in 2023. A replication site was established in mid-2024 to support independent data collection using the same preregistered protocol. At the time of submission, data collection for the patient cohort is advanced but ongoing, with recruitment and control data collection still in progress. All experimental procedures and analysis plans were preregistered in advance of data collection (see [16]), and any deviations or updates to the protocol are being documented transparently within the OSF record (https://osf.io/4rn85). We anticipate completion of remaining data collection and final analyses within the projected study timeline. Consistent with INTREPID consortium practices, any protocol amendments are coordinated across sites and theory leads (Karl Friston, Cyriel Pennartz, and Giulio Tononi) and recorded with justification to preserve transparency and interpretability of the adversarial design.

### Dissemination plans

Results from this experimental protocol are expected to be disseminated through peer-reviewed publication, either as a stand-alone article or in combination with related experiments conducted within the INTREPID adversarial collaboration. Findings may also be presented at scientific conferences in poster or talk format.
