# Supplementary material for: Protocol for measuring the effects of cortical scotoma on visual space perception
Source: PLoS One. 2026 Nov 11;21(11):e0352036. doi: 10.1371/journal.pone.0352036 (PMC13754219; doi:10.1371/journal.pone.0352036)
Supplement: Supplementary file 1 [file pone.0352036.s002.docx]

Thank you for submitting your manuscript to PLOS ONE. After careful consideration, we feel that it has merit but does not fully meet PLOS ONE’s publication criteria as it currently stands. Therefore, we invite you to submit a revised version of the manuscript that addresses the points raised during the review process.

==============================
This is a clear and promising protocol testing whether cortical scotomas alter perceived visual space or instead remove stimulus content within an otherwise preserved spatial field. The 2IFC design and PSE-based outcome are appropriate and interpretable.

My main concern is that some procedural and analytic decisions should be specified more operationally, including scotoma mapping, stimulus placement, block length, data acceptability, task pooling, and the interpretation of heterogeneous outcomes. Although the manuscript states that the study was preregistered and identifies the global PSE as the primary outcome, it would be useful to clarify which decisions were fixed in the original preregistration, which remain flexible, and which have been subsequently clarified or updated, especially because data collection is already underway.

Please state more operationally how PSE shifts will be interpreted as evidence for contraction, no contraction, or mixed outcomes. Please also specify the planned criteria for scotoma mapping, stimulus placement, fixation quality, exclusions, acceptable psychometric fits, lapse-rate handling, and additional blocks. In addition, please justify the planned pooling of the two-dot, three-dot, and contour tasks, as these may tap partly different spatial judgments. Finally, please specify whether and how relevant patient-level variables will be recorded and considered, including scotoma geometry, lesion location, time since lesion, residual vision, subjective scotoma experience, prior rehabilitation or testing, and fixation stability.
I also encourage the authors to carefully consider the reviewers’ comments.

Overall, the study is suitable for publication as a protocol after these clarifications.

We thank the editor for pointing out these issues, most of which intersect with specific comments made by the reviewers. All of these points have been addressed to some degree in revisions to the text, in combination with changes due to other comments from the reviewers.

On the items brought up by the Academic Editor:

- Scotoma mapping: more information is provided about stopping rules (which are ‘soft’, since the shape and extent of a VFD may be extremely different from one participant to the next), clarifying the goals of the experimenter in capturing the shape of the VFD, and with objective minimum numbers for the perimetry stages. Ultimately we feel the exact parameters of the manual perimetry should be left to the judgment of the experimenters, to best adapt to what is sure to be wide variation in scotoma presentations. (paragraph starting around line 286)
- Stimulus placement: some rewording should make clear that the stimuli are placed so as to be “well out of danger” of falling into the margins of a scotoma, while still forcing “scotoma-crossing” of stimulus configurations. (paragraph starting around line 322)
- Block length: as clarified in the text, block length is determined by the number of repeats of the staircases that compose the block; blocks will be added only according to time and stamina considerations (if there is time for another block, and does the participant seem up to it). (paragraph starting around line 370)
- Data acceptability: There are two points to address here-

First is “block rejection”. A little more information was included to explain that when the task is done properly, even with rather few trials (as few as 24), the shape of a psychometric function should be clearly visible on quick inspection. If instead the hitrate is “flat”, independent of stimulus value, it is a sign of a data collection problem (which should be immediately addressed before more trials are run), and such blocks are not to be included in data analysis. It is conceivable that a subject will fail after multiple blocks to produce “good” data, though we have not encountered such a case with patients we have tested, or in other similar studies in our lab (as specified in inclusion criteria, we shall exclude candidate participants with cognitive or other impairments that would seem to preclude their being able to respond normally in a 2IFC task). (again, paragraph around line 370)

Second is “data quality”. Our policy is to include all trials where a subject was able to produce coherent (non-rejected) data. If a subject’s data is exceedingly noisy (due to very poor discrimination, i.e. an inordinately shallow psychometric function slope, or due to high lapse rate), this naturally translates to poorly-constrained estimates of the PSE (very broad CIs, or failure to distinguish a PSE shift from none at all in a putative model-selection procedure, such as is now suggested in the text). Such data will thus contribute less to any judgments regarding presence or absence of VFD effects on PSEs. (see Data Analysis)

- Task pooling: Discussion has been extended to clarify the motivations here. Different tasks were included in the plan so as not to put “all our eggs in one basket” – if there are effects of VFD on distance judgment, perhaps they are picked-out by some tasks better than others. Since the preregistration did not distinguish outcomes of the different tasks (this is now said clearly in the text), the primary analysis should be agnostic to task type (hence the trial-pooling strategy) – however, if consistent differences are observed, these will be of great interest and may aid in constraining hypotheses regarding causes and mechanisms underlying whatever effects are observed. (see Data Analysis)
- Patient characteristics: The only etiological aspect to the preregistration of the study was that the participants should have lesion to “early visual cortex”. However, as should now be clearer in the text, we will certainly gather and record as much information as we can about each participant’s unique history – including conditions of the lesion (stroke, injury, surgery), time since injury, participation in rehabilitation, idiosyncracies of the VFD (sharp or ragged margins; reports of interior experiences of probes; etc). (see new paragraph around line 272)
  - (Beyond this, we also plan to collect detailed post-experiment debriefing interviews with patients about their experiences, although this was not included in the preregistered plan (it is an “add on” study conceived after the original proposal) and so the interview plan is not detailed in this protocol paper.)

Journal Requirements:

When submitting your revision, we need you to address these additional requirements.

1. Please ensure that your manuscript meets PLOS ONE's style requirements, including those for file naming. The PLOS ONE style templates can be found at

https://journals.plos.org/plosone/s/file?id=wjVg/PLOSOne_formatting_sample_main_body.pdf and

<https://journals.plos.org/plosone/s/file?id=ba62/PLOSOne_formatting_sample_title_authors_affiliations.pdf>

2. Thank you for stating the following financial disclosure:
“Templeton World Charity Foundation (TWCF)”

Please state what role the funders took in the study. If the funders had no role, please state: "The funders had no role in study design, data collection and analysis, decision to publish, or preparation of the manuscript."
If this statement is not correct you must amend it as needed.
Please include this amended Role of Funder statement in your cover letter; we will change the online submission form on your behalf.

done

3. We note that you have a patent relating to material pertinent to this article. Please provide an amended statement of Competing Interests to declare this patent (with details including name and number), along with any other relevant declarations relating to employment, consultancy, patents, products in development or modified products etc. Please confirm that this does not alter your adherence to all PLOS ONE policies on sharing data and materials, as detailed online in our guide for authors http://journals.plos.org/plosone/s/competing-interests by including the following statement: "This does not alter our adherence to PLOS ONE policies on sharing data and materials.” If there are restrictions on sharing of data and/or materials, please state these. Please note that we cannot proceed with consideration of your article until this information has been declared.

This information should be included in your cover letter; we will change the online submission form on your behalf.

done

4. Please provide a complete Data Availability Statement in the submission form, ensuring you include all necessary access information or a reason for why you are unable to make your data freely accessible. If your research concerns only data provided within your submission, please write "All data are in the manuscript and/or supporting information files" as your Data Availability Statement.

done

5. One of the noted authors is a group or consortium “TWCF-INTREPID consortium”. In addition to naming the author group, please list the individual authors and affiliations within this group in the acknowledgments section of your manuscript. Please also indicate clearly a lead author for this group along with a contact email address.

done

6. Please include your full ethics statement in the ‘Methods’ section of your manuscript file. In your statement, please include the full name of the IRB or ethics committee who approved or waived your study, as well as whether or not you obtained informed written or verbal consent. If consent was waived for your study, please include this information in your statement as well.

done

7. If the reviewer comments include a recommendation to cite specific previously published works, please review and evaluate these publications to determine whether they are relevant and should be cited. There is no requirement to cite these works unless the editor has indicated otherwise.

done

8. Please review your reference list to ensure that it is complete and correct. If you have cited papers that have been retracted, please include the rationale for doing so in the manuscript text, or remove these references and replace them with relevant current references. Any changes to the reference list should be mentioned in the rebuttal letter that accompanies your revised manuscript. If you need to cite a retracted article, indicate the article’s retracted status in the References list and also include a citation and full reference for the retraction notice.

done

6. Review Comments to the Author

Please use the space provided to explain your answers to the questions above and, if applicable, provide comments about issues authors must address before this protocol can be accepted for publication. You may also include additional comments for the author, including concerns about research or publication ethics.

You may also provide optional suggestions and comments to authors that they might find helpful in planning their study.


(Please upload your review as an attachment if it exceeds 20,000 characters)

Reviewer #1: This protocol presents a unique approach to investigate how spatial representation is altered in patients with scotomas arising from cortical injury. This patient population is being employed as a model system in which to compare three theories of consciousness, with various predictions made as to the outcome of the present experiment depending on which theory is most accurate. Overall, this is a very exciting protocol, with interesting implications for philosophy, vision science, and translations outcomes. My primary concerns for this protocol stem from gaps in the literature that should be considered prior to forming any conclusions from the proposed studies.

Major concerns:
The present protocol is missing references to key literature that may be informative to task design, expectations, and interpretation. Dilks et al., 2007 conducted a similar experiment presenting objects across the border of the blind field and observed altered perception of the object (DOI: 10.1523/JNEUROSCI.2650-07.2007). Several groups, including Bridge, Huxlin, and Smirnakis have reported residual V1 activity extending into the blind field, which would have major implications for the interpretation of any detected distortions. Furthermore, the cortically impaired field is known to be non-uniform, with many patients reported residual visual abilities within perimetrically defined blind fields, which can fluctuate day to day (see Riddoch 1917, Weiskrantz 1974, Campion 1983). Thus, treating the scotoma has a region of blindness may lead to incorrect conclusions regarding the various cognitive theories being tested. Finally, the visual system is known to adapt in numerous ways to the presence of cortical damage. The proposed predictions from each theory appears not to account for these changes. For example, increased connectivity between subcortical and extrastriate regions may allow portions of the visual field to behave just as it would if V1 were to remain intact.

We thank the reviewer for this comment. We have added text to better recognize the complexity of the visual scotoma landscape; it is indeed true that there have been other studies of distortion in proximity to scotomas (including cortical scotomas, retinal and other pre-cortical defects, and artificial/simulated scotomas). In addition, yes, cortical scotomas are not monolithic and may present very differently in different patients: sometimes the defect is extremely deep, sometimes there is patchy residual vision to certain target sizes/intensities/etc; sometimes there is a degree of “blindsight” – sometimes the margin is sharp (as is typical along the vertical meridian of a hemianopia or quadrantanopia), sometimes ragged and very irregular (as typical along the horizontal boundary of a quadrantanopia). We added discussion at two points to make clear that these variables may contribute to the heterogeneity of the participant sample (see responses to the Editor, above).

Minor concerns:
Mapping of the scotoma is said to be done “The experimenter queries the participant verbally (“do you see the probe?”). The participant verbally responds “yes” or “no” to indicate whether the probe was seen…”. Patients with cortical vision loss are often reported to be overly conservative with their response criteria, and so they may possess percepts of the probe that they do not consider “seeing”. The experimenters should take care when asking for patient reports to ensure they receive the most accurate feedback they can.

We added some detail to the perimetry procedure to address this point. In our experience, two general regimes of response criterion are likely to be encountered: in the best cases, patients will confidently say *no* when asked if they see a probe within the defect, and when it is moved into seeing field, they will report not only that they see it, but that it becomes visible abruptly. In these cases, the question is simply : “do you see the probe”, and the criterion “yes or no” is sufficient. However, it is not unusual to encounter a more difficult case, where the patient evinces some uncertainty, or asks for clarification, because they see something unclear or intermediate, especially in the ragged margin of the defect. In these cases, we try to push the subject towards a more “liberal” criterion: “tell me if you see *something*”. (see new discussion of perimetry response criterion, around lines 272). Any such discussions about features of the scotoma are noted and attached to the subject’s data.

The statement “the strength of support depends also on the confidence with which predictions were made" suggest patient confidence and/or metacognitive outcomes will be assessed, but there are no details regarding this collection provided in the methods.

This is a misunderstanding – ‘confidence’ here referred to the confidence of the theory leads as expressed in their preregistered predictions, *not* in any metacognitive measures collected (there are none in this study). This was reworded to avoid confusion or ambiguity. [line 583]

Minor edits:
“a range of possible contractions or . If any positions fall within the scotoma or outside the display,”

fixed

Reviewer #2: the manuscript describes the protocol of a pre-registered study. The study concept has been peer reviewed by the TWCF. Furthermore the hypotheses have been preregistered. the paper describes coherently the protocol of the study and needs no further peer review.

Much appreciated, thank you.

Reviewer #3: This is an interesting, seemingly well designed study protocol for experiments proposed as part of INTREPID, an adversarial collaboration funded by the Templeton World Charities Foundation. It aims to test competing theories of conscious visual processing in humans with damage to early visual cortex. The goal here is to ascertain if visual space is actively constructed by higher-level processes (presumably intact in the patient population of interest), or whether it is defined by the intrinsic connectivity of early visual cortex (which is damaged in the population of interest). The study at 2 sites (University of Wisconsin-Madison and Schepens Eye Research Institute -SERI- in Boston) is ongoing but not yet completed. Ethical approval has been obtained at both sites, demonstrating that they have met required ethical standards.

The study protocol appears overall technically sound, contains some (but not all) of the information needed for replication and should be able to generally address the questions posed.

Nonetheless, there are concerns, as well as a lot of missing information that should be addressed, as the missing information especially, diminishes the ability to replicate the study:

1. Consideration of key prior work is missing from the Introduction: there has been prior study of spatial distortions experienced across scotomas by patients with insults to early visual cortex. A notable example includes Dilks et al., J Neurosci 2007. This should be cited and discussed.

In two places we have added some review of relevant literature (we had aimed to reserve this talk for the research paper, but in fact it is good to admit some of it here). First, in the perimetry section (beginning around line 272), there is added discussion of the variability of qualities of experience (or non-experience) in a cortical scotoma, recognizing the likelihood of inhomegenous field loss and quasi-visual perception in cortical scotomas; second, in the discussion, we recount previous studies that have touched on distortions produced by various types of scotoma (pre-cortical, cortical, and artificial), citing Dilks, Safran, and others. We believe this added depth helps to support the rationale underlying the current study.

2. Another missing consideration is the subjective experience of the patients. Blind fields are not uniform. In addition, some patients experience them as completely filled-in with surrounding information – like a large blind spot. Others see jagged edges or recognize them as an area of blurriness in their visual field, but with preserved ability to perceive objects moving through them. Will these percepts be recorded and used to categorize patients to better interpret their data? It seems that the 2 examples of patients I just described above would be predicted to generate quite different results.

See previous comment.

3. Nowhere in this manuscript (not even in Eligibility Criteria and Recruitment) can I find information about the planned sample sizes and their justifications. Patients with occipital lobe damage are not that common. Restricting the study to those who have quadrantanopias or scotomas as proposed here will significantly decrease this sample size further. Without knowing exactly what the variance in the PSE might be, it is really impossible for me to gauge whether the study protocol will actually generate interpretable results and on which of the 3 tasks proposed. Given that the study has already accumulated patient data (as stated in Status and Timeline and given their many publications, detailed on the study web site), perhaps this information can be computed and provided.

This is indeed a real issue. Our target N (planned sample size) was based on discussions of feasibility – a “best case” estimate of what we could achieve in recruitment in the study period, after talking with clinical collaborators at both sites. We hope to achieve these numbers but they are *not* based in any form of power analysis – we felt this would be inadvisable given the subject population (generally older and inexperienced subjects who may have concomitant issues making good performance in a 1-shot psychophysics experiment difficult to obtain). However, we are confident that the relative simplicity of the task (it is a time-tested traditional 2IFC design) and our block-to-block procedure for identifying “bad data” (blocks of trials where the subject did not properly perform the task), in addition to normal statistical methods for weighing estimates of PSE shift based on data quality (“noisy” data will yield estimates with wide confidence intervals), will ensure that most or all participants will provide interpretable data for the project.

In addition to this, there is the fact that prior studies of related phenomena (in particular those by Dilks’ and Safran’s groups) typically focused on just a few subjects (1 in the 2007 Dilks paper; just 2 in the important Safran studies) – here we hope to gather data on dozens of patients. Thus, while we aim to be able to adjudicate effects on a single-subject basis, we will have the further ability to adjudicate over a distribution of such effects.

Finally, consider the experiment simulation shown in Figure 3. This illustrates a plausible “normal case” – a subject that can provide a few hundred trials, with an insignificant lapse rate, and a discrimination threshold around 10%, yielding an expected stdev in PSE estimation of around s=0.038. Although we cannot report results in this protocol, I can state that based on results so far (which we cannot include here due to the prospective nature of the Protocol format), these numbers are a realistic reflection of what can be obtained in the patient population. In this case, as now stated in the text (paragraph starting at line 492), an effect as small as plus or minus 6% would be picked up in 95% of tests.

a. I attempted to sign in to OSF and see if I could access the pre-registered study record to find this information, but I was unable to do so.

Apologies, that link was outdated. Though it was technically possible to wade through a thicket of osf pages to get to the actual prereg document, there is now a direct link to the current document: <https://osf.io/4rn85>. This was updated in the text. It should not be necessary to sign in to view the document.

4. For the SERI labs, will participants also be screened for their prior enrollment in visual rehabilitation studies? SERI has a long history of performing studies with prism lenses and other testing/training protocols. Such practice could alter participants’ priors and expertise with estimating spatial relationships inside and across their scotomas. This potential confound should be considered, discussed, and managed in the proposed study.

Yes, a patient’s participation in rehabilitation will be recorded along with other meaningful information (time since injury; form of injury; age; etc). This is mentioned explicitly in the text now (line 174).

5. Related to the issue of prior experience by the patients after their cortical damage, for interpreting PSE estimation results with respect to the NREP theory, it seems that time since the insult, the age at which the insult was sustained (childhood versus adulthood) and how much opportunity the patient has had to recalibrate themselves using their intact other senses and motor functions would both be important. Will these be measured/recorded? Will patients be sorted according to those factors? Are they part of the inclusion/exclusion criteria and if so, where are those criteria detailed? Such information should be made readily available as part of this protocol.

Yes, see previous comment.

6. For Tasks 1 and 2, it seems important to ensure that the 2 or 3 dots (respectively) are all contained within the same hemifield of vision – either that affected or unaffected by the brain damage. If one dot is placed in the intact hemifield and the other(s) in the affected hemifield, we are then dealing with cross-hemisphere estimations. Will this be controlled and how?

In quadrantanopic defects in particular, it is unavoidable that the scotoma-spanning stimulus (pairs or trios of dots) will fall on opposite sides of the vertical midline, and that the ‘normal field’ comparison stimulus will be entirely to one side of the midline. Such defects cannot be spanned otherwise. Ideally the test and standard stimuli would be at perfectly symmetrical positions, and while this is possible in cases of paracentral scotoma (where the defect can be spanned vertically, and where the defect may not fully abut the vertical midline), it is not possible in quadrantanopia. This is now noted explicitly in the protocol (around line 317), along with the fact that we will endeavor to make stimuli as symmetrical as possible.

That said, while there is plenty of evidence for hemifield differences in visual ability, and for dips in certain forms of visual performance in the vicinity of the vertical meridian (e.g. Carrasco et al have recently done a lot in this field), we do not have any reason to expect that *crossing* the midline should yield a special sort of result in comparison with merely being in close proximity to the midline. In the end, given the likely wide variety in the layout of field defects we will encounter, we should have many different stimulus layouts, some which offend in this respect more than others, and any such interaction (involving the relative proximity of test and standard stimulus dots to the vertical midline) can be investigated post-hoc.

7. For Task 3, the shape size comparison, will the shape always be larger than the scotoma? How much larger and why? And how exactly will the scotoma be defined? Using perimetry described in Figure 1? This is apparently not the case, which is confusing, as that is likely more precise than the 2- or 3-dot tasks.

Yes. The procedure is explained in a little more detail in the Task 3 section. The procedure involves fitting an ellipse to the *test points*, ie. the positions where the task 1 and 2 dots would fall. These are already, by design, placed beyond the scotoma boundary. On top of this fitted ellipse, a random oscillation (producing bumpy protrusions) is added, expanding the radius at all points by between +10% and +40%. These values were chosen by pre-testing the stimulus-generation algorithm, to ensure (in the case of some model paracentral scotomas drawn from previous publications by Safran et al, and from the Peli group) that the shape contours would never cross through the scotoma (the +10% floor seemed to do the job), nor fall off the computer display.

8. There are no recommended checklists provided as SI files and while the authors have registered this project on OSF, I do not have permission to access any of the materials.

Our understanding is that the checklist recommendations apply to completed research studies, so we have not attached any checklists. If this is in error and we need to attach something further, it should be caught by the handling editor.

9. It is unclear if this work is considered a clinical trial or not – please make this clear in the text.

It is not, this is now explicitly stated in the Recruitment section.

10. How will the authors interpret results that show some patients to exhibit performance consistent with one theory and others consistent with another theory? This is not detailed, and is pertinent to inter-individual variability on multiple fronts, some of which I raised in Pts 2, 3, 4 and 5.

To be honest: this remains to be seen. We (the authors responsible for running the study, led by Haun and Peli, respectively) will run this as a “normal study” and endeavor to present the results as objectively as possible. On top of our work, the adversarial theory leads will then hammer out interpretations of the results. Ideally, results will be unambiguous and one theory will be able to straightforwardly “claim victory”, but in practice things are rarely so simple.

Nevertheless, this comment did prompt us to expand a bit on how data will be interpreted, splitting off a “connecting results to adversarial predictions” section below ‘Data analysis’ - and how different outcomes might support (or not) different theories.
