# Supplementary material for: Protocol for measuring the effects of cortical scotoma on visual space perception
Source: PLoS One. 2026 Nov 11;21(11):e0352036. doi: 10.1371/journal.pone.0352036 (PMC13754219; doi:10.1371/journal.pone.0352036)
Supplement: Peer review history

# Peer review: Decision Letter 0

**DOI:** 10.1371/journal.pone.0352036.r001

**Authors:** Giulio Contemori

2 Jul 2026

Dear Dr. Haun,

Thank you for submitting your manuscript to PLOS ONE. After careful consideration, we feel that it has merit but does not fully meet PLOS ONE’s publication criteria as it currently stands. Therefore, we invite you to submit a revised version of the manuscript that addresses the points raised during the review process.

==============================

This is a clear and promising protocol testing whether cortical scotomas alter perceived visual space or instead remove stimulus content within an otherwise preserved spatial field. The 2IFC design and PSE-based outcome are appropriate and interpretable.

My main concern is that some procedural and analytic decisions should be specified more operationally, including scotoma mapping, stimulus placement, block length, data acceptability, task pooling, and the interpretation of heterogeneous outcomes. Although the manuscript states that the study was preregistered and identifies the global PSE as the primary outcome, it would be useful to clarify which decisions were fixed in the original preregistration, which remain flexible, and which have been subsequently clarified or updated, especially because data collection is already underway.

Please state more operationally how PSE shifts will be interpreted as evidence for contraction, no contraction, or mixed outcomes. Please also specify the planned criteria for scotoma mapping, stimulus placement, fixation quality, exclusions, acceptable psychometric fits, lapse-rate handling, and additional blocks. In addition, please justify the planned pooling of the two-dot, three-dot, and contour tasks, as these may tap partly different spatial judgments. Finally, please specify whether and how relevant patient-level variables will be recorded and considered, including scotoma geometry, lesion location, time since lesion, residual vision, subjective scotoma experience, prior rehabilitation or testing, and fixation stability.

Overall, the study is suitable for publication as a protocol after these clarifications.

==============================

Please submit your revised manuscript by Jul 17 2026 11:59PM. If you will need more time than this to complete your revisions, please reply to this message or contact the journal office at plosone@plos.org. When you're ready to submit your revision, log on to https://www.editorialmanager.com/pone/ and select the 'Submissions Needing Revision' folder to locate your manuscript file.

- A letter that responds to each point raised by the academic editor and reviewer(s). You should upload this letter as a separate file labeled 'Response to Reviewers'.
- A marked-up copy of your manuscript that highlights changes made to the original version. You should upload this as a separate file labeled 'Revised Manuscript with Track Changes'.
- An unmarked version of your revised paper without tracked changes. You should upload this as a separate file labeled 'Manuscript'.

If you would like to make changes to your financial disclosure, please include your updated statement in your cover letter. Guidelines for resubmitting your figure files are available below the reviewer comments at the end of this letter.

If applicable, we recommend that you deposit your laboratory protocols in protocols.io to enhance the reproducibility of your results. Protocols.io assigns your protocol its own identifier (DOI) so that it can be cited independently in the future. For instructions see: https://journals.plos.org/plosone/s/submission-guidelines#loc-laboratory-protocols. Additionally, PLOS ONE offers an option for publishing peer-reviewed Lab Protocol articles, which describe protocols hosted on protocols.io. Read more information on sharing protocols at https://plos.org/protocols?utm_medium=editorial-email&utm_source=authorletters&utm_campaign=protocols.

As the corresponding author, your ORCID iD is verified in the submission system and will appear in the published article. PLOS supports the use of ORCID, and we encourage all coauthors to register for an ORCID iD and use it as well. Please encourage your coauthors to verify their ORCID iD within the submission system before final acceptance, as unverified ORCID iDs will not appear in the published article. *Only*  the individual author can complete the verification step; PLOS staff *cannot*  verify ORCID iDs on behalf of authors.

We look forward to receiving your revised manuscript.

Kind regards,

Giulio Contemori, Ph.D.

Academic Editor

PLOS One

Journal Requirements:

When submitting your revision, we need you to address these additional requirements.

1. Please ensure that your manuscript meets PLOS ONE's style requirements, including those for file naming. The PLOS ONE style templates can be found at

https://journals.plos.org/plosone/s/file?id=wjVg/PLOSOne_formatting_sample_main_body.pdf and

https://journals.plos.org/plosone/s/file?id=ba62/PLOSOne_formatting_sample_title_authors_affiliations.pdf

2. Thank you for stating the following financial disclosure:

“Templeton World Charity Foundation (TWCF)”

Please state what role the funders took in the study. If the funders had no role, please state: "The funders had no role in study design, data collection and analysis, decision to publish, or preparation of the manuscript."

If this statement is not correct you must amend it as needed.

Please include this amended Role of Funder statement in your cover letter; we will change the online submission form on your behalf.

3. We note that you have a patent relating to material pertinent to this article. Please provide an amended statement of Competing Interests to declare this patent (with details including name and number), along with any other relevant declarations relating to employment, consultancy, patents, products in development or modified products etc. Please confirm that this does not alter your adherence to all PLOS ONE policies on sharing data and materials, as detailed online in our guide for authors http://journals.plos.org/plosone/s/competing-interests by including the following statement: "This does not alter our adherence to PLOS ONE policies on sharing data and materials.” If there are restrictions on sharing of data and/or materials, please state these. Please note that we cannot proceed with consideration of your article until this information has been declared.

This information should be included in your cover letter; we will change the online submission form on your behalf.

4. Please provide a complete Data Availability Statement in the submission form, ensuring you include all necessary access information or a reason for why you are unable to make your data freely accessible. If your research concerns only data provided within your submission, please write "All data are in the manuscript and/or supporting information files" as your Data Availability Statement.

5. One of the noted authors is a group or consortium “TWCF-INTREPID consortium”. In addition to naming the author group, please list the individual authors and affiliations within this group in the acknowledgments section of your manuscript. Please also indicate clearly a lead author for this group along with a contact email address.

6. Please include your full ethics statement in the ‘Methods’ section of your manuscript file. In your statement, please include the full name of the IRB or ethics committee who approved or waived your study, as well as whether or not you obtained informed written or verbal consent. If consent was waived for your study, please include this information in your statement as well.

7. If the reviewer comments include a recommendation to cite specific previously published works, please review and evaluate these publications to determine whether they are relevant and should be cited. There is no requirement to cite these works unless the editor has indicated otherwise.

8. Please review your reference list to ensure that it is complete and correct. If you have cited papers that have been retracted, please include the rationale for doing so in the manuscript text, or remove these references and replace them with relevant current references. Any changes to the reference list should be mentioned in the rebuttal letter that accompanies your revised manuscript. If you need to cite a retracted article, indicate the article’s retracted status in the References list and also include a citation and full reference for the retraction notice.

[Note: HTML markup is below. Please do not edit.]

Reviewers' comments:

Reviewer's Responses to Questions

**Comments to the Author**

1. Does the manuscript provide a valid rationale for the proposed study, with clearly identified and justified research questions?

Reviewer #1: Yes

Reviewer #2: Yes

Reviewer #3: Yes

**********

2. Is the protocol technically sound and planned in a manner that will lead to a meaningful outcome and allow testing the stated hypotheses?

Reviewer #1: Yes

Reviewer #2: Yes

Reviewer #3: Partly

**********

3. Is the methodology feasible and described in sufficient detail to allow the work to be replicable?

Reviewer #1: Yes

Reviewer #2: Yes

Reviewer #3: No

**********

4. Have the authors described where all data underlying the findings will be made available when the study is complete?

The PLOS Data policy

Reviewer #1: Yes

Reviewer #2: Yes

Reviewer #3: Yes

**********

5. Is the manuscript presented in an intelligible fashion and written in standard English?

Reviewer #1: Yes

Reviewer #2: Yes

Reviewer #3: Yes

**********

Please use the space provided to explain your answers to the questions above and, if applicable, provide comments about issues authors must address before this protocol can be accepted for publication. You may also include additional comments for the author, including concerns about research or publication ethics.

You may also provide optional suggestions and comments to authors that they might find helpful in planning their study.

Reviewer #1: This protocol presents a unique approach to investigate how spatial representation is altered in patients with scotomas arising from cortical injury. This patient population is being employed as a model system in which to compare three theories of consciousness, with various predictions made as to the outcome of the present experiment depending on which theory is most accurate. Overall, this is a very exciting protocol, with interesting implications for philosophy, vision science, and translations outcomes. My primary concerns for this protocol stem from gaps in the literature that should be considered prior to forming any conclusions from the proposed studies.

Major concerns:

The present protocol is missing references to key literature that may be informative to task design, expectations, and interpretation. Dilks et al., 2007 conducted a similar experiment presenting objects across the border of the blind field and observed altered perception of the object (DOI: 10.1523/JNEUROSCI.2650-07.2007). Several groups, including Bridge, Huxlin, and Smirnakis have reported residual V1 activity extending into the blind field, which would have major implications for the interpretation of any detected distortions. Furthermore, the cortically impaired field is known to be non-uniform, with many patients reported residual visual abilities within perimetrically defined blind fields, which can fluctuate day to day (see Riddoch 1917, Weiskrantz 1974, Campion 1983). Thus, treating the scotoma has a region of blindness may lead to incorrect conclusions regarding the various cognitive theories being tested. Finally, the visual system is known to adapt in numerous ways to the presence of cortical damage. The proposed predictions from each theory appears not to account for these changes. For example, increased connectivity between subcortical and extrastriate regions may allow portions of the visual field to behave just as it would if V1 were to remain intact.

Minor concerns:

Mapping of the scotoma is said to be done “The experimenter queries the participant verbally (“do you see the probe?”). The participant verbally responds “yes” or “no” to indicate whether the probe was seen…”. Patients with cortical vision loss are often reported to be overly conservative with their response criteria, and so they may possess percepts of the probe that they do not consider “seeing”. The experimenters should take care when asking for patient reports to ensure they receive the most accurate feedback they can.

The statement “the strength of support depends also on the confidence with which predictions were made" suggest patient confidence and/or metacognitive outcomes will be assessed, but there are no details regarding this collection provided in the methods.

Minor edits:

“a range of possible contractions or . If any positions fall within the scotoma or outside the display,”

Reviewer #2: the manuscript describes the protocol of a pre-registered study. The study concept has been peer reviewed by the TWCF. Furthermore the hypotheses have been preregistered. the paper describes coherently the protocol of the study and needs no further peer review.

Reviewer #3: This is an interesting, seemingly well designed study protocol for experiments proposed as part of INTREPID, an adversarial collaboration funded by the Templeton World Charities Foundation. It aims to test competing theories of conscious visual processing in humans with damage to early visual cortex. The goal here is to ascertain if visual space is actively constructed by higher-level processes (presumably intact in the patient population of interest), or whether it is defined by the intrinsic connectivity of early visual cortex (which is damaged in the population of interest). The study at 2 sites (University of Wisconsin-Madison and Schepens Eye Research Institute -SERI- in Boston) is ongoing but not yet completed. Ethical approval has been obtained at both sites, demonstrating that they have met required ethical standards.

The study protocol appears overall technically sound, contains some (but not all) of the information needed for replication and should be able to generally address the questions posed.

Nonetheless, there are concerns, as well as a lot of missing information that should be addressed, as the missing information especially, diminishes the ability to replicate the study:

1. Consideration of key prior work is missing from the Introduction: there has been prior study of spatial distortions experienced across scotomas by patients with insults to early visual cortex. A notable example includes Dilks et al., J Neurosci 2007. This should be cited and discussed.

2. Another missing consideration is the subjective experience of the patients. Blind fields are not uniform. In addition, some patients experience them as completely filled-in with surrounding information – like a large blind spot. Others see jagged edges or recognize them as an area of blurriness in their visual field, but with preserved ability to perceive objects moving through them. Will these percepts be recorded and used to categorize patients to better interpret their data? It seems that the 2 examples of patients I just described above would be predicted to generate quite different results.

3. Nowhere in this manuscript (not even in Eligibility Criteria and Recruitment) can I find information about the planned sample sizes and their justifications. Patients with occipital lobe damage are not that common. Restricting the study to those who have quadrantanopias or scotomas as proposed here will significantly decrease this sample size further. Without knowing exactly what the variance in the PSE might be, it is really impossible for me to gauge whether the study protocol will actually generate interpretable results and on which of the 3 tasks proposed. Given that the study has already accumulated patient data (as stated in Status and Timeline and given their many publications, detailed on the study web site), perhaps this information can be computed and provided.

a. I attempted to sign in to OSF and see if I could access the pre-registered study record to find this information, but I was unable to do so.

4. For the SERI labs, will participants also be screened for their prior enrollment in visual rehabilitation studies? SERI has a long history of performing studies with prism lenses and other testing/training protocols. Such practice could alter participants’ priors and expertise with estimating spatial relationships inside and across their scotomas. This potential confound should be considered, discussed, and managed in the proposed study.

5. Related to the issue of prior experience by the patients after their cortical damage, for interpreting PSE estimation results with respect to the NREP theory, it seems that time since the insult, the age at which the insult was sustained (childhood versus adulthood) and how much opportunity the patient has had to recalibrate themselves using their intact other senses and motor functions would both be important. Will these be measured/recorded? Will patients be sorted according to those factors? Are they part of the inclusion/exclusion criteria and if so, where are those criteria detailed? Such information should be made readily available as part of this protocol.

6. For Tasks 1 and 2, it seems important to ensure that the 2 or 3 dots (respectively) are all contained within the same hemifield of vision – either that affected or unaffected by the brain damage. If one dot is placed in the intact hemifield and the other(s) in the affected hemifield, we are then dealing with cross-hemisphere estimations. Will this be controlled and how?

7. For Task 3, the shape size comparison, will the shape always be larger than the scotoma? How much larger and why? And how exactly will the scotoma be defined? Using perimetry described in Figure 1? This is apparently not the case, which is confusing, as that is likely more precise than the 2- or 3-dot tasks.

8. There are no recommended checklists provided as SI files and while the authors have registered this project on OSF, I do not have permission to access any of the materials.

9. It is unclear if this work is considered a clinical trial or not – please make this clear in the text.

10. How will the authors interpret results that show some patients to exhibit performance consistent with one theory and others consistent with another theory? This is not detailed, and is pertinent to inter-individual variability on multiple fronts, some of which I raised in Pts 2, 3, 4 and 5.

**********

what does this mean?). If published, this will include your full peer review and any attached files.

If you choose “no”, your identity will remain anonymous but your review may still be made public.

**Do you want your identity to be public for this peer review?**  For information about this choice, including consent withdrawal, please see our Privacy Policy

Reviewer #1: **Yes:**  Matthew Cavanaugh

Reviewer #2: No

Reviewer #3: No

**********

[NOTE: If reviewer comments were submitted as an attachment file, they will be attached to this email and accessible via the submission site. Please log into your account, locate the manuscript record, and check for the action link "View Attachments". If this link does not appear, there are no attachment files.]

To ensure your figures meet our technical requirements, please review our figure guidelines: https://journals.plos.org/plosone/s/figures

You may also use PLOS’s free figure tool, NAAS, to help you prepare publication quality figures: https://journals.plos.org/plosone/s/figures#loc-tools-for-figure-preparation.

NAAS will assess whether your figures meet our technical requirements by comparing each figure against our figure specifications.

# Author comment: Author response to Decision Letter 1

**DOI:** 10.1371/journal.pone.0352036.r002

5 Aug 2026

Our responses to the reviewers and the editor are contained in a separate document.

Attachment Submitted filename: responsetoreviewers.docx

# Peer review: Decision Letter 1

**DOI:** 10.1371/journal.pone.0352036.r003

**Authors:** Giulio Contemori

15 Sep 2026

Protocol for measuring the effects of cortical scotoma on visual space perception

PONE-D-26-16887R1

Dear Dr. Haun,

We’re pleased to inform you that your manuscript has been judged scientifically suitable for publication and will be formally accepted for publication once it meets all outstanding technical requirements.

Within one week, you’ll receive an e-mail detailing the required amendments. When these have been addressed, you’ll receive a formal acceptance letter and your manuscript will be scheduled for publication.

An invoice will be generated when your article is formally accepted. Please note, if your institution has a publishing partnership with PLOS and your article meets the relevant criteria, all or part of your publication costs will be covered. Please make sure your user information is up-to-date by logging into Editorial Manager at Editorial Manager® and clicking the ‘Update My Information' link at the top of the page. For questions related to billing, please contact billing support.

If your institution or institutions have a press office, please notify them about your upcoming paper to help maximize its impact. If they’ll be preparing press materials, please inform our press team as soon as possible -- no later than 48 hours after receiving the formal acceptance. Your manuscript will remain under strict press embargo until 2 pm Eastern Time on the date of publication. For more information, please contact onepress@plos.org.

Kind regards,

Giulio Contemori, Ph.D.

Academic Editor

PLOS One

Additional Editor Comments (optional):

The authors have satisfactorily addressed the comments raised during the previous round of review. The revised manuscript is clear and the reviewers have no further concerns or requests. I am therefore pleased to recommend acceptance of the manuscript in its current form.

Reviewers' comments:

Reviewer's Responses to Questions

**Comments to the Author**

1. Does the manuscript provide a valid rationale for the proposed study, with clearly identified and justified research questions?

Reviewer #1: Yes

Reviewer #3: Yes

**********

2. Is the protocol technically sound and planned in a manner that will lead to a meaningful outcome and allow testing the stated hypotheses?

Reviewer #1: Yes

Reviewer #3: Yes

**********

3. Is the methodology feasible and described in sufficient detail to allow the work to be replicable?

Reviewer #1: Yes

Reviewer #3: Yes

**********

4. Have the authors described where all data underlying the findings will be made available when the study is complete?

The PLOS Data policy

Reviewer #1: Yes

Reviewer #3: Yes

**********

5. Is the manuscript presented in an intelligible fashion and written in standard English?

Reviewer #1: Yes

Reviewer #3: Yes

**********

Please use the space provided to explain your answers to the questions above and, if applicable, provide comments about issues authors must address before this protocol can be accepted for publication. You may also include additional comments for the author, including concerns about research or publication ethics.

You may also provide optional suggestions and comments to authors that they might find helpful in planning their study.

Reviewer #1: The authors have sufficiently addressed all of my prior comments. I have no further edits or concerns.

Reviewer #3: The authors have carefully addressed the reviewers' and editorial board member's comments. I have no further suggestions or requests.

**********

what does this mean?). If published, this will include your full peer review and any attached files.

If you choose “no”, your identity will remain anonymous but your review may still be made public.

**Do you want your identity to be public for this peer review?**  For information about this choice, including consent withdrawal, please see our Privacy Policy

Reviewer #1: **Yes:**  Matthew Cavanaugh

Reviewer #3: No

**********

# Editor report: Acceptance letter

**DOI:** 10.1371/journal.pone.0352036.r004

**Authors:** Giulio Contemori

PONE-D-26-16887R1

PLOS One

Dear Dr. Haun,

I'm pleased to inform you that your manuscript has been deemed suitable for publication in PLOS One. Congratulations! Your manuscript is now being handed over to our production team.

At this stage, our production department will prepare your paper for publication. This includes ensuring the following:

* All references, tables, and figures are properly cited

* All relevant supporting information is included in the manuscript submission,

* There are no issues that prevent the paper from being properly typeset

You will receive further instructions from the production team, including instructions on how to review your proof when it is ready. Please keep in mind that we are working through a large volume of accepted articles, so please give us a few days to review your paper and let you know the next and final steps.

Lastly, if your institution or institutions have a press office, please let them know about your upcoming paper now to help maximize its impact. If they'll be preparing press materials, please inform our press team within the next 48 hours. Your manuscript will remain under strict press embargo until 2 pm Eastern Time on the date of publication. For more information, please contact onepress@plos.org.

You will receive an invoice from PLOS for your publication fee after your manuscript has reached the completed accept phase. If you receive an email requesting payment before acceptance or for any other service, this may be a phishing scheme. Learn how to identify phishing emails and protect your accounts at https://explore.plos.org/phishing.

If we can help with anything else, please email us at customercare@plos.org.

Thank you for submitting your work to PLOS One and supporting open access.

Kind regards,

PLOS One Editorial Office Staff

on behalf of

Dr. Giulio Contemori

Academic Editor

PLOS One
